# Testing the Strength and Vulnerability Integration (SAVI) Model of Emotion in a Sample of Latinx Adults.

**DOI:** 10.1093/geroni/igaf122.3277

**Published:** 2025-12-31

**Authors:** Robert Kennison, Jaiwei Xiang

**Affiliations:** California State University, Los Angeles, Los Angeles, California, United States; California State University, Los Angeles, Los Angeles, California, United States

## Abstract

The strength and vulnerability integration (SAVI) model identifies two pathways that affect emotional well-being in aging (Turk Charles, 2010). The strengths pathway theorizes that increases in well-being result from age-related changes in perspective (e.g., expansive time horizon), which enhance strengths like emotion regulation (e.g., cognitive reappraisal). The vulnerabilities pathway posits that age-related threats to well-being include greater physiological reactivity to stress. This vulnerability can be offset by avoiding or de-escalating stressful events. These predicted relationships were examined in survey data that included SAVI-proximate variables collected from a predominately Latinx (74.7%) sample of adults aged 18-89 (n = 573). Latent variable modeling was performed. First, latent measures of expansive time horizon, cognitive reappraisal, and well-being were tested in a measurement model that fit the data. Second, measures of age, number of reported health conditions and concern for the consequences of stress were integrated into the model that replicated the pathways identified by SAVI, producing an acceptable fit (CFI=.94, TLI=.93, RMSEA=.06). For the strengths pathway, advancing age predicted less-expansive time horizons (B=-.02), which predicted greater use of cognitive reappraisal (B=.27) and greater well-being (B=.18); cognitive reappraisal predicted greater well-being (B=.23). For the vulnerabilities pathway, advancing age predicted number of health conditions (B=.02) and greater well-being (B=.01); health conditions predicted concern for the consequences of stress (B=.33), which predicted poorer well-being (B=-.04). These results are broadly consistent with the SAVI model. Importantly, differences between Latinx and non-Latinx groups were not observed, suggesting that the SAVI model translates to cultures typically understudied in aging research.

